# Assessing the Effect of Sequencing Depth and Sample Size in Population Genetics Inferences

**DOI:** 10.1371/journal.pone.0079667

**Published:** 2013-11-18

**Authors:** Matteo Fumagalli

**Affiliations:** Department of Integrative Biology, University of California, Berkeley, California, United States of America; Natural History Museum of Denmark, University of Copenhagen, Denmark

## Abstract

Next-Generation Sequencing (NGS) technologies have dramatically revolutionised research in many fields of genetics. The ability to sequence many individuals from one or multiple populations at a genomic scale has greatly enhanced population genetics studies and made it a data-driven discipline. Recently, researchers have proposed statistical modelling to address genotyping uncertainty associated with NGS data. However, an ongoing debate is whether it is more beneficial to increase the number of sequenced individuals or the per-sample sequencing depth for estimating genetic variation. Through extensive simulations, I assessed the accuracy of estimating nucleotide diversity, detecting polymorphic sites, and predicting population structure under different experimental scenarios. Results show that the greatest accuracy for estimating population genetics parameters is achieved by employing a large sample size, despite single individuals being sequenced at low depth. Under some circumstances, the minimum sequencing depth for obtaining accurate estimates of allele frequencies and to identify polymorphic sites is 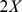, where both alleles are more likely to have been sequenced. On the other hand, inferences of population structure are more accurate at very large sample sizes, even with extremely low sequencing depth. This all points to the conclusion that under various experimental scenarios, in cost-limited population genetics studies, large sample sizes at low sequencing depth are desirable to achieve high accuracy. These findings will help researchers design their experimental set-ups and guide further investigation on the effect of protocol design for genetic research.

## Introduction

One primary aim of population genetics studies is understanding the relative role of neutral and selective forces in shaping the overall genetic diversity of populations. This is often nowadays achieved by investigating the amount and patterns of genetic variation across multiple samples at a large genomic scale. However, until recently, studies relied on the analysis of sequencing data for short genomic regions or for a limited number of candidate genes, or on the analysis of genotypes from sparse Single Nucleotide Polymorphism (SNP) data. While the former approach produces accurate inferences, it targets a small fraction of the genome, and the latter provides insights at the genome-wide level but can be prone to considerable ascertainment bias, which has been shown to inflate certain results [1]. The main obstacle precluding more extensive analyses relates to high experimental costs.

In the last few years, new high-throughput DNA sequencing technologies have allowed researchers to generate large amounts of genetic data. Such Next-Generation-Sequencing (NGS) technologies are now a common tool in population genetics [2], medical genetics [3] and other genetic disciplines [4]. While NGS technologies may differ in their protocols, the data produced by them all have similar general characteristics [5]: short fragments of sequenced DNA known as “reads” are mapped to a reference genome or *de novo* aligned. The data on which all downstream analyses are performed typically consists of a collection of mapped reads covering a particular genomic position, with associated base and mapping quality scores. Each site in the alignment can be covered by a variable number of reads (a feature called “sequencing depth”). Individual genotypes are then inferred from the allelic state of the reads covering the site of interest (a procedure called “genotype calling”), while “SNP calling” refers to the process of identifying which sites are polymorphic in the sample, that is, have more than 1 base type at the site.

Sequencing depth is an important characteristic of the data. Genotypes called for sites with higher depth are likely to be more accurate, while lower sequencing depth leads to a non-negligible amount of genotyping uncertainty [6]. Since SNP calling proceeds from genotype calling, sequencing depth influences the detection of variable sites. Factors such as sequencing and mapping errors add to the uncertainty in genotype and SNP calling from NGS data.

Recently proposed methods that employ statistical models accommodate this uncertainty by using genotype likelihoods and have been successfully applied to empirical datasets (e.g. [7]). Such methods include those used for estimating allele frequencies at a single site [8]–[10] or jointly across multiple sites [9], [11], [12], mutation rates [13], and several population genetics summary statistics and parameters [11], [12], [14]–[17].

NGS technologies are a powerful tool for investigating the evolutionary forces that shape genomes. Many summary statistics used for analysing demography, natural selection, and population structure, are derived from estimates of nucleotide variation across multiple individuals [18]. The number of segregating sites and the allele frequencies at these sites are among the most important features of the data from an evolutionary perspective, and are the basis of commonly used neutrality tests [19]–[22].

Genetic structure is another extremely important feature of populations that can be discerned from population genetics data. Realising population structure provides insights into demographic history [23], and has practical use in clinical association studies [24]. Principal Component Analysis (PCA) is a long-standing statistical tool for examining genetic structure among individuals because it reduces highly-dimensional genetic data into a map of uncorrelated components based on the covariance among genotypes [25].

Population genetics inferences will become more accurate with greater sample sizes, that is, with more individuals representing a particular population. However, at a fixed research budget, sequencing more samples will lower the per-sample sequencing depth, and, as a consequence, increase the genotype uncertainty. Similarly, higher sequencing coverage will decrease genotyping uncertainty, but will also restrict the analysis to a smaller sample of individuals, which may be a poor representation of the genomic variation of the entire population. Recent whole-genome sequencing projects have adopted both the former [26]–[29] and the latter strategy [30].

It is therefore appealing to investigate the relationship between the accuracy in estimating within- and between-populations genetic variation and the sequencing experimental design. The sequencing strategy can easily be modelled in terms of the number of sequenced samples and the per-sample sequencing depth. Despite the extensive use of NGS data in population genetics, the effect on the accuracy of estimates of genetic variation by different sequencing strategies has yet to be thoroughly quantified.

Through simulation of sequencing data and by using state-of-the-art statistical methods for estimating genetic variation from NGS data, I quantified the accuracy of estimating the number of segregating sites, nucleotide diversity, allele frequencies, and population structure under a wide range of sequencing scenarios. These results will help researchers optimise their sequencing experiments.

## Results and Discussion

### Estimating Nucleotide Diversity

Extensive simulations were performed to evaluate the accuracy of estimating nucleotide diversity under various sequencing conditions and fixed experimental budget. The cost is assumed to be proportional to the total sequencing depth, which is a function of the number of individuals and target size. Therefore, experiments with equal cost will have equal total sequencing depth. Although this may be not strictly true, this assumption is a reasonable generalization given current NGS technologies.

A total of 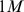, and 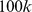, sites of DNA sequencing data were simulated at an average per-sample sequencing depth of 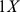, 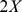, 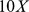, and 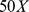. Corresponding sample sizes were 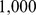, 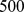, 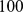, and 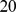 diploid individuals, so that the product of the sample size and sequencing coverage was the same across scenarios. The standardised bias for estimates of the number of segregating sites (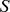) and the expected heterozygosity (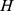), between the case of known genotypes for all 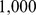 individuals and the case of unknown genotypes for all or a fraction of individuals, was calculated. Sequence data was divided into 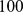 independent windows and the bias in the estimates for the population genetics statistics was computed for each region separately (see Methods).

**Figure 1:**
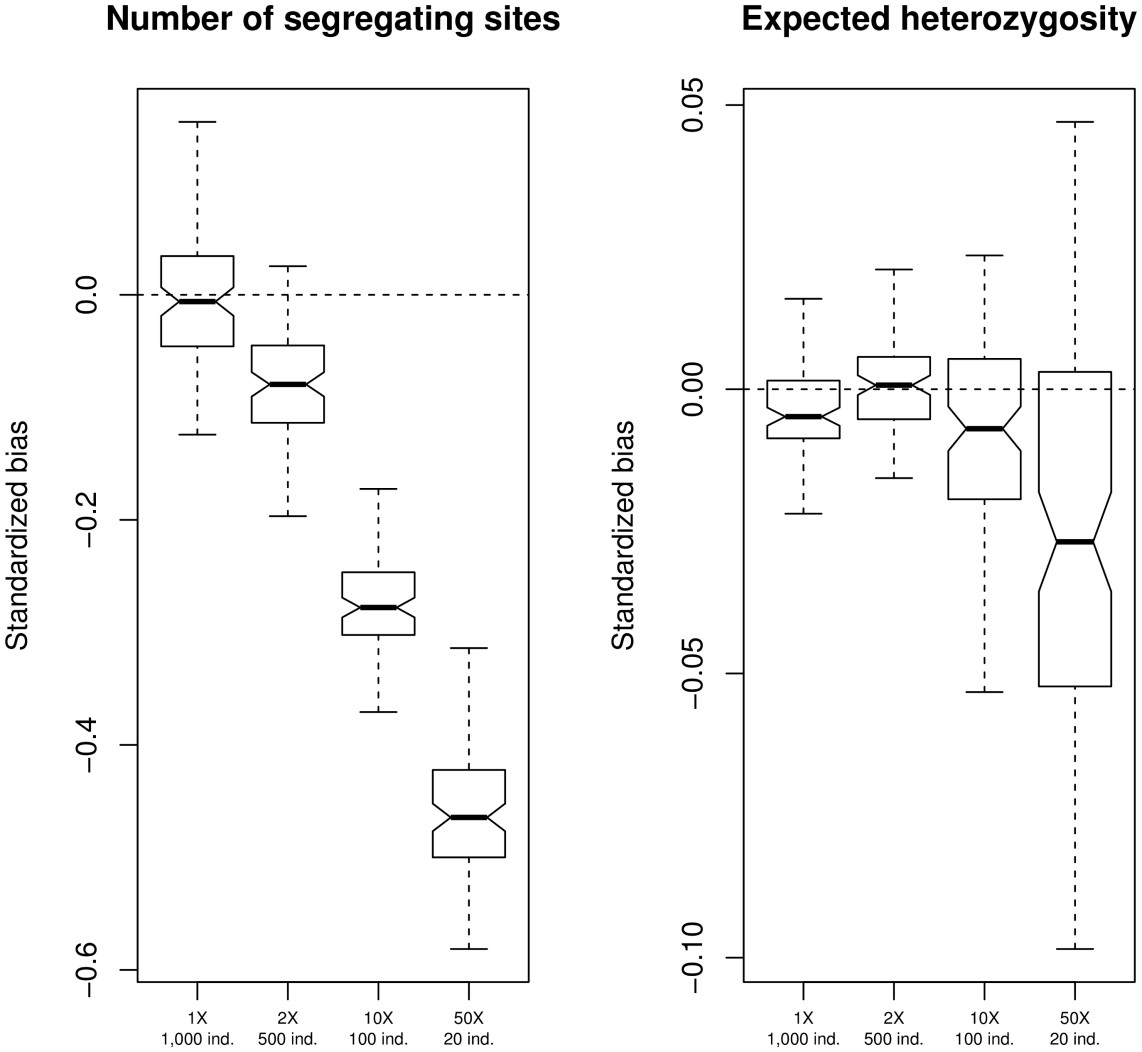
Nucleotide diversity estimation. Bias in the estimate of the number of segregating sites (left panel) and the expected heterozygosity (right panel) under different experimental scenarios. Sequencing depths are 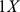, 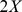, 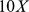, and 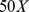 and the corresponding sample sizes are 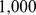, 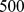, 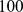, and 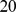 individuals. I simulated 100 regions of 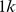 independent sites, with a probability of each site being variable in the population equal to 0.1.

The highest accuracy for estimating the number of segregating sites was achieved at a larger sample size despite the lower sequencing depth (Figure 1). In all scenarios, the true number of segregating sites in the population was underestimated, but this error approaches 0 in the 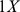 coverage condition. The error rapidly increases at higher sequencing depth and lower sample size. At 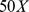 coverage for 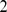
0 individuals, the number of segregating sites is underestimated by up to 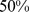.

Secondly, estimates for the expected heterozygosity from simulated sequencing data were compared to estimates of heterozygosity with known genotypes. Heterozygosity is a function of allele frequency (see Methods). Heterozygosity is severely underestimated at high sequencing depth and small sample size, while an approximately unbiased estimate is achieved at 2X coverage for 500 sequenced individuals (Figure 1). Similar results are observed when simulating a larger number of sites with lower variability (Figure S1) or lower sequencing error rate (Figure S2).

When sequencing depth is low, under-estimating 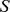 and 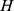 can be attributed to the smaller probability of sequencing the alternate allele from heterozygotes. On the other hand, when sample sizes are small, 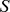 and 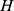 are under-estimated due to heterozygotes not being sampled. The results clearly show that, despite lower sequencing depths, larger sample sizes produce more accurate estimates of population genetics variation. Furthermore, increasing sample size affords greater accuracy for detecting nucleotide diversity outliers, with a sequencing depth of 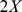 for 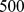 individuals giving the highest correlation between true and estimated values (Table S1).

Under a simulated population expansion model (e.g. like in humans [31]), estimates of nucleotide diversity at high sequencing depth and small sample size were even more biased than under the constant population size model (Figure S3). Under population expansion, the site frequency spectrum is skewed towards low frequency variants, which are not captured well when sequencing only a small number of individuals. This effect increases the error when estimating nucleotide diversity.

The number of segregating sites and nucleotide diversity were also estimated under conditions in which genotype proportions deviated from Hardy-Weinberg Equilibrium (HWE) due to inbreeding. Specifically, an individual inbreeding coefficient of 0.3 was used for the simulations (see Methods). This inbreeding scenario is representative of highly structured populations, self-pollinating plants, and domesticated species. The highest accuracy in estimating the number of segregating sites and nucleotide diversity was achieved when employing many samples at low sequencing depth (Figure S4). The general decrease in accuracy when estimating average heterozygosity is caused by violation of the HWE assumption upon which the method used to estimate heterozygosity relies [11]. Further studies to generalise models for estimating allele frequencies from sequencing data when HWE does not hold are strongly encouraged [32].

Sequencing a large number of samples at the trade-off of lower individual coverage represents the optimal design for accurately inferring population nucleotide diversity. Under some scenarios, the highest accuracy for estimating the expected heterozygosity, which is a function of the sample allele frequency, is achieved at 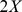 sequencing depth, where both alleles are more likely to have been sequenced, versus 1X coverage. These findings are robust to different assumptions of population demography and mating system.

### Identifying Polymorphic Sites

SNP calling is the procedure for identifying which sites are polymorphic in a sample, and hence in the population from which the sample was drawn. The False Positive (FP) and False Negative (FN) rates, and Precision and Recall values (see Methods) were calculated under all experimental scenarios in order to assess SNP calling accuracy. FP measures how many non variable sites are misidentified as being polymorphic, while FN measures how many SNPs are not identified as being variable. Precision and Recall measure the proportion of relevant calls for FP and FN, respectively (see Methods). High values of Precision and Recall, and low values of FP and FN are desirable.

Precision and Recall values for SNP calling under different scenarios are shown in Table 1. A site was considered to be a SNP if its probability of being variable exceeded a given threshold, which was dynamically chosen to minimise the difference between the true and estimated number of variable sites in the entire population. This approach is not realistic outside of simulations, but guarantees an optimal equilibrium between FP and FN (i.e. their sum is approximately constant). As expected, Precision increases with higher sequencing depth. For instance, at 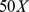, Precision is 1, indicating that all called SNPs are truly polymorphic. On the other hand, as sequencing depth increases and the sample size is reduced, Recall values decrease. This reflects the inability to call variable sites when heterozygous individuals are not sequenced. The highest Recall is obtained at 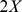 sequencing depth for 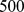 individuals, at which point the Precision is comparable to a scenario that uses depth of 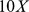 for 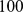 individuals. Similar results are obtained when filtering out sites with low total sequencing depth (see Methods) (Table S2). As expected, when identifying polymorphisms solely at the sequenced sample level, as opposed to the population level, both Precision and Recall increase with higher sequencing depth (Table S3).

**Table 1:** SNP calling Precision and Recall.

Sequencing depth	Sample size	Precision	Recall
1X	1,000	0.737 (0.0437)	0.749 (0.0472)
2X	500	0.778 (0.0461)	0.771 (0.0446)
10X	100	0.779 (0.0441)	0.725 (0.0408)
50X	20	1 (0)	0.540 (0.0582)

Precision and Recall values for detecting polymorphic sites at different scenarios of sequencing depth and sample size. Values are averaged across 100 different replicates and standard deviations are reported in parentheses.

**Figure 2:**
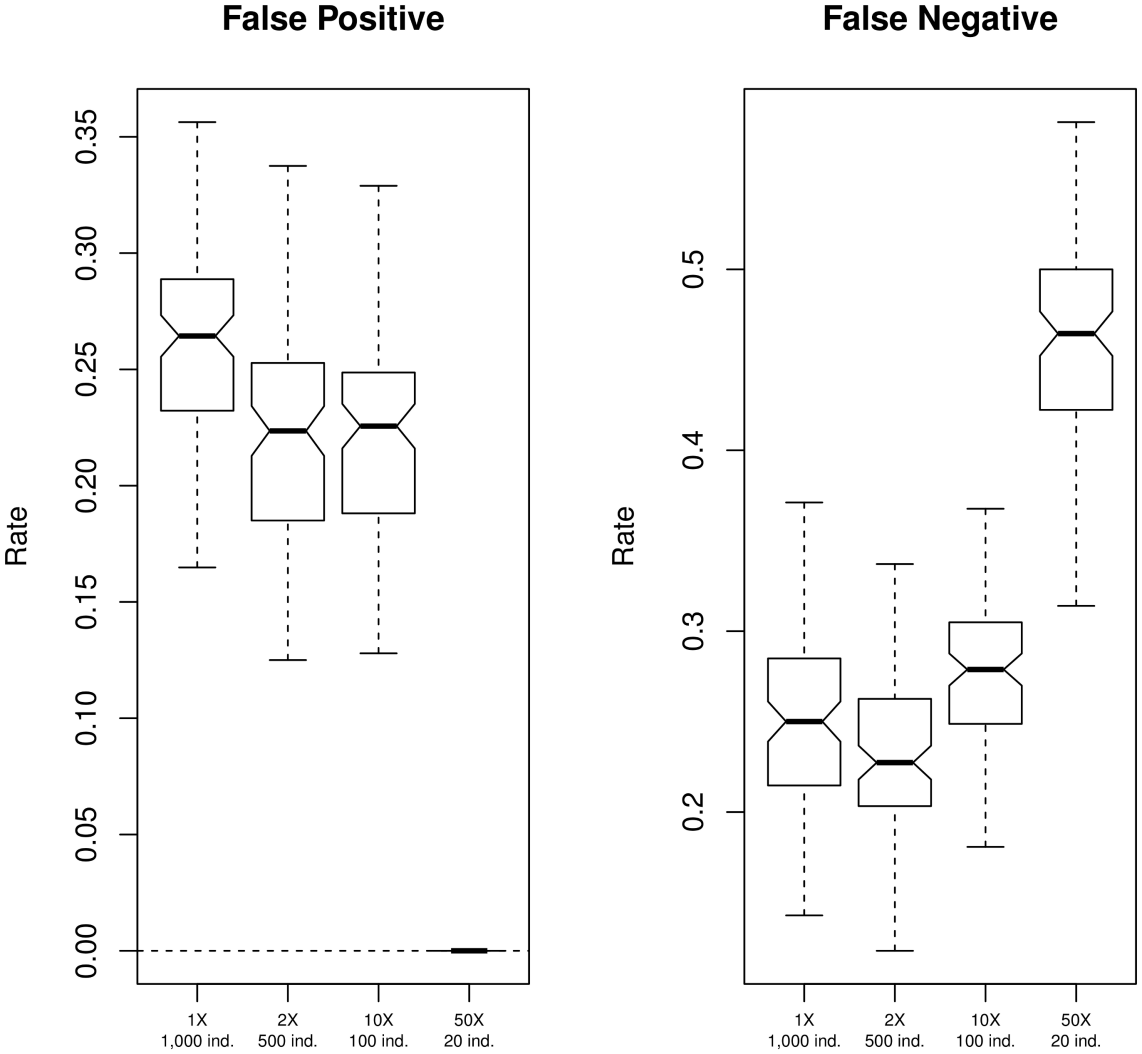
SNP calling accuracy. False Positive and False Negative rates in the identification of polymorphic sites under different experimental scenarios. Simulations were performed as described in Figure 1. Sites were identified as polymorphic if their probability of being variable was above a threshold, chosen to minimise the difference between the true and the estimated number of SNPs (see Methods).

These trends, as well as the distribution of FP and FN rates, are similar across all windows (Figure 2). The FP rate is higher in cases of low sequencing depth, especially at 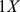, while it is 0 at 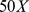. The opposite effect is observed for FN rates, which are higher at 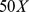; specifically, almost 50% of true SNPs are not detected. The median FN rate at 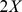 is the lowest among all tested experimental conditions (Figure 2). Similar results are obtained when simulating genotype frequencies not in HWE (Figure S5), and for a population under an expansion model, although, in the latter case, 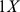, 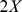, and 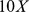 designs show comparable levels of accuracy (Figure S6).

Then, I performed SNP calling by assigning polymorphisms if the probability of being variable was greater than a fixed threshold, namely 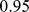. This strategy is similar to common practice. For all scenarios, FP rates drop to 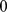, while FN rates increase and median values are above 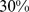 (Figure S7). Indeed, SNPs are called only if high confidence is achieved. Similar results are obtained in case of population expansion (Figure S8) and deviation from HWE (Figure S9). A less stringent threshold for SNP calling reduces the FN rate (Figure S10), while a more stringent cut-off increases FN values (Figure S11).

SNP calling accuracy was also assessed when confined to common variants, defined as sites with a minor allele frequency greater than 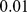, which is equivalent to an absolute frequency of 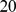 chromosomes, out of 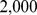 total chromosomes, bearing the alternate allele. SNPs were called if their probability of being variable was greater than 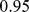. Notably, FN rates have a median equal to 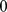 in 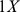 and 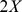 cases, while it is close to 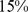 for 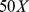 (Figure S12). Accuracy increases if rare variants, which are more likely not to be identified, are ignored. Similar results were obtained in the cases of population expansion (Figure S13) and deviation from HWE (Figure S14).

The results suggest that SNP calling is greatly influenced by the joint effect of sample size and sequencing depth. Generally, high sequencing depth provides greater Precision, while greater Recall is obtained with higher sample size. However, calling SNPs using a common strategy based on the probability of each site being variable reduces FP rates to 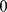 for all scenarios. Nevertheless, FN rates are always greater than 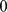 with small sample sizes. A sequencing depth lower than 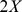 precludes accurate identification of variable sites because of the lower chance of sequencing both alleles at the individual level. These findings are robust to different assumptions for population size changes and deviation from HWE. As expected, most of the misidentified true variable sites have low minor allele frequency. Indeed, SNP calling on common variants produces FN rates close to 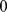 for all sequencing configurations except at the lowest sample size.

### Predicting Population Structure

I simulated sequencing data for multiple sub-populations to test the accuracy of inferring population structure under different sequencing depth and sample size conditions. Specifically, I simulated 3 populations of 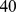 individuals each, at different levels of genetic differentiation, with the per-sample sequencing depth set to 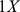, 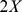, 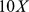, and 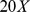, and corresponding sample sizes of 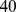, 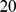, 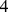, and 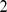 individuals from each of the 3 populations, so that total sequencing depth was equal across designs. One hundred simulations were performed under each sequencing scenario to account for variation in individual sub-sampling (see Methods).

The first 2 Principal Components (PCs) in a Principal Components Analysis (PCA) were used to train a predictive model of population structure on a 2-dimensional grid through a Support Vector Machine (SVM) technique. For each cell of the grid, I assigned a population based on the model trained from known genotypes and from sequencing data. The proportion of mislabelled cells, where the model from sequencing data predicts a different population than the model trained by known genotypes (see Methods), was recorded. Accuracy in predicting population structure is then inversely proportional to the fraction of mislabelled cells, and can be quantified on an arbitrary grid.

**Figure 3:**
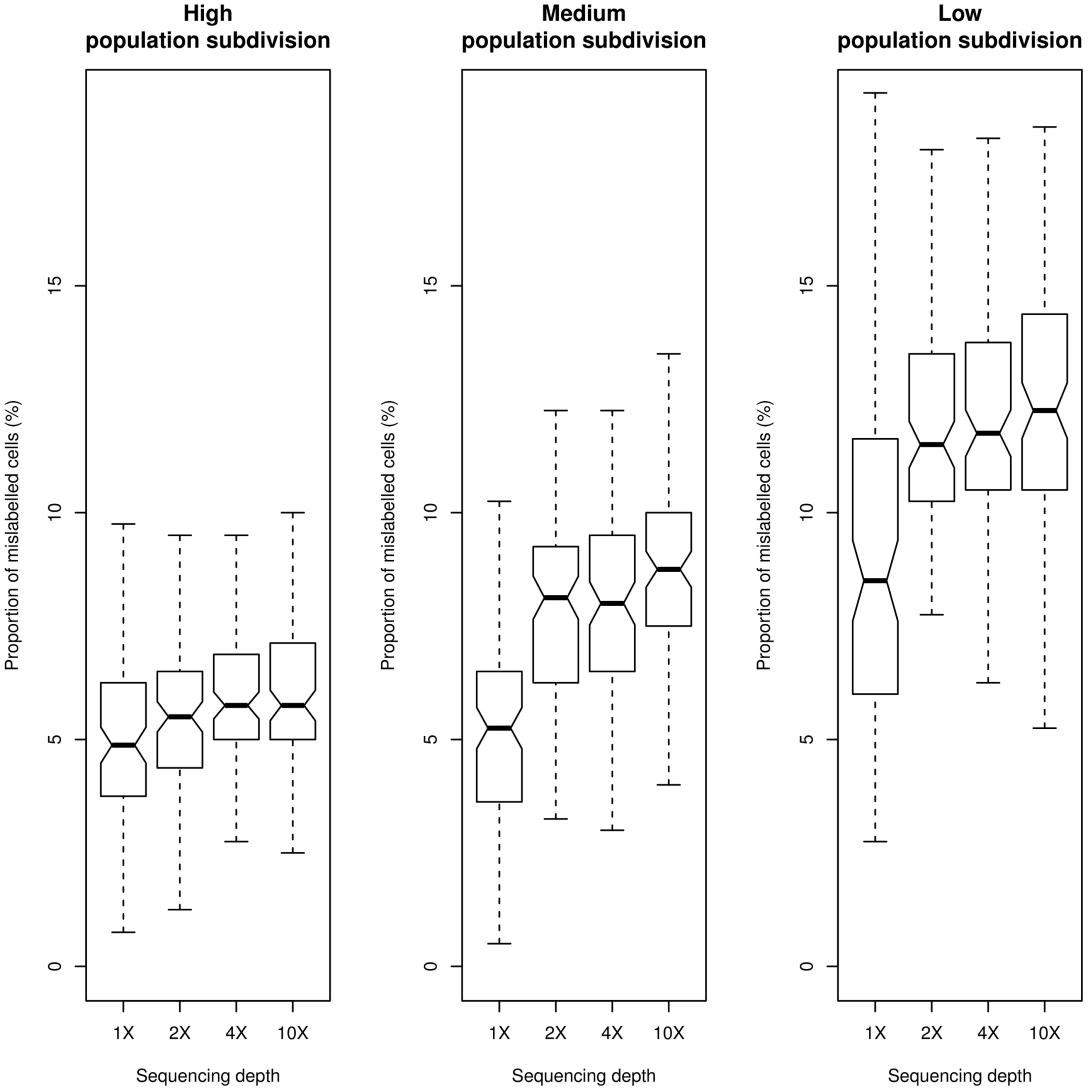
Population structure inference accuracy. Accuracy of population structure inference, measured as the proportion of cells over a 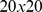 grid where sub-populations have been wrongly assigned from sequencing data compared to the case of known genotypes for all individuals (see Methods). Sequencing depths are 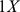, 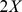, 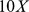, and 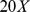 and the corresponding sample sizes are 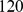, 60, 12, and 6 individuals. I simulated 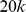 independent sites, with a probability of each site being variable in the population equal to 0.1. Populations were simulated with high genetic subdivision (left panel, 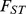 0.4 and 0.1), medium genetic subdivision (mid panel, 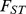 0.3 and 0.05), low genetic subdivision (right panel, 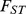 0.1 and 0.02).

Results show that the design with 1X sequencing depth and 40 individuals sampled from each population achieves the highest accuracy in predicting population structure (Figure 3). This effect is more pronounced for cases involving low-to-medium genetic differentiation between populations. Under these conditions, sequencing less samples produces 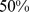 more mislabelled cells, on average, than using all individuals at very low sequencing depth. Similar results were obtained with a less dense grid (Figure S15), and when simulating only variable sites (Figure S16). The latter finding suggests that monomorphic sites do not influence predictions even at low sequencing depth.

**Figure 4:**
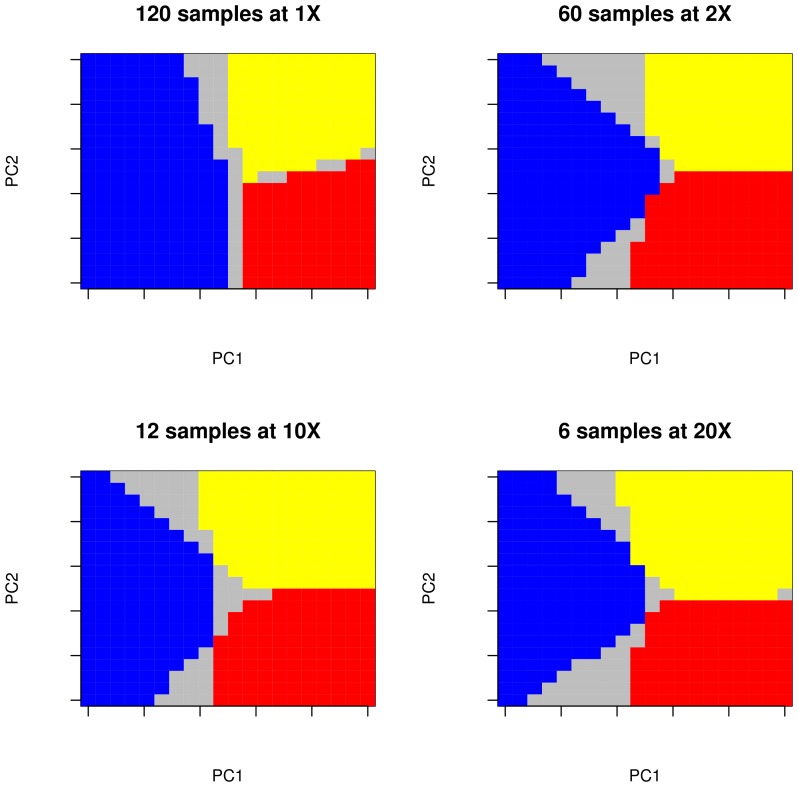
Population structure prediction. Population structure predicted over a 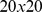 grid for a single replicate under different experimental scenarios. Simulations were performed as described in Figure 3, in the case of low genetic subdivision. Grey cells represent locations where a different sub-population was predicted to be located from sequencing data compared to the case of known genotypes of all individuals. These particular replicates show a proportion of mislabelled cells equal to be the medium of the distribution. Note that replicates are not the same across the different tested scenarios.

To illustrate the overall trend in distinguishing population structure, the inferred population structure was plotted over a grid for a single simulation, assuming low genetic differentiation among populations. For each scenario, a simulation having accuracy equal to the median for the entire distribution was chosen to represent the overall behaviour. Figure 4 shows that most of the mislabelled cells lie on the borders between populations. As already seen in Figure 3, an experimental design in which all individuals have been sequenced at low depth provides the greatest accuracy for predicting population structure.

## Conclusions

For this study, extensive simulations were performed under a wide range of sequencing designs to test the joint effect of sequencing depth and sample size on population genetics inferences. The results suggest that at a fixed sequencing budget, it is desirable to sequence a large number of individuals, at the cost of reducing the per-sample sequencing depth.

To estimate allele frequencies and identify polymorphic sites, sequencing the largest possible sample size with at least a per-sample sequencing depth of 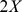 is recommended. Similarly, population structure is more accurately inferred at low depth with large sample sizes, and even at depth as low as 1X if a large enough sample size is used.

It is also important to consider that state-of-the-art statistical methods to estimate genetic variation from NGS data were used [11]. These approaches, based on genotype likelihoods, provide superior estimates to methods employing strict genotype calling [11], [17], [33], and therefore should be adopted in all population genetics studies using low-medium coverage sequencing data.

I believe that this study will assist researchers in their experimental design. The approach for testing the effect of experimental conditions on population genetics inferences used in this study can be extended to other fields in genomics and medical genetics.

## Methods

### Simulating Sequencing Data

Sequencing data was extensively simulated to assess the accuracy of estimating nucleotide variation and population structure under different experimental scenarios. Simulated individual genotypes were assigned assuming Hardy-Weinberg Equilibrium (HWE), and an inbreeding coefficient of 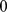 or 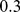, given an ancestral population allele frequency. This ancestral allele frequency was drawn from an exponential distribution, which is proportional to the expected allele frequency distribution under a standard neutral diffusion model [34]. To mimic the genomic effect of population expansion, I artificially skewed the expected allele frequency distribution towards low frequency variants by squaring, and then normalising, the values in the site frequency spectrum. The number of reads at each locus for each individual was drawn from a Poisson distribution [10], [35]. Sequencing errors were randomly and uniformly introduced among reads at rates of 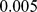 and 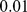, which are comparable to empirical error rates [26], [27]. The probability of a site being polymorphic in the population was set to 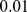, 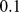, and 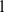.

For analyses related to estimating within-population nucleotide diversity, the individual per-site mean sequencing depths (the average number of mapped reads) were set to 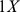, 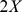, 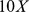 or 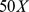 for different corresponding sample sizes in order to achieve a constant total sequencing depth of 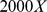 across all individuals. I simulated 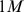, and 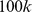, independent diallelic sites for 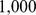 individuals. The information content produced by these simulations is comparable to the output of current high-throughput sequencing machines.

To simulate population structure, sub-population allele frequencies were drawn from a Beta distribution [36] with mean equal to the ancestral population allele frequency [37]. To simulate data from 3 populations, allele frequencies for two sub-populations were drawn as just described and the first of these frequencies was assigned to sub-population 1. The second allele frequency was assigned as the ancestral allele frequency for sub-populations 2 and 3. To model variable degrees of genetic sub-division among populations in the Beta distribution [36], different values of 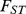, a common measure of population genetics differentiation [38], were assumed. I simulated population structure with low (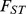 values of 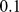 and 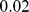), medium (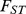 values of 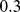 and 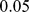), and high (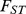 values of 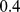 and 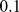) genetic sub-division.

For population structure analyses, I simulated 3 populations of 40 individuals each, and a total of 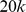 independent diallelic sites. Then, 40, 20, 4, or 2 individuals per population were sampled, with corresponding sequencing depth of 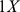, 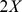, 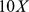, and 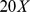, resulting in a total sequencing depth of 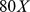 per population. Given that individuals can be sampled in many different combinations, I performed 100 replicates for each experimental scenario.

### Computing Nucleotide Diversity from Sequencing Data

Accuracy for estimating nucleotide diversity from sequencing data was assessed by first dividing all 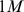, and 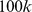, simulated sites into 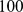 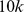, and 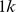, non-overlapping windows. For each window, I calculated the proportion of segregating sites (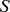) as the fraction of variable sites in the sample, and the expected heterozygosity (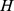). In the case of known genotypes, these quantities can be easily calculated across 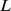 sites as:
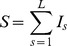
(1)where 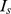 is an indicator function equal to 1 when at least one individual is heterozygous at site 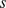, and 0 otherwise, and
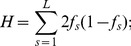
(2)where 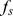 is the reference allele frequency for a site, 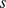, in the sample.

When genotypes are unknown, they must be inferred from the mapped sequence read data. Current studies use genotype likelihoods to ultimately call genotypes when necessary. Genotype likelihoods are a function of both base calls and quality scores and are proportional to the probability of the observed data given a certain genotype, for a given site in an individual [12], [39]. Bayesian methods have been proposed to calculate the posterior probability 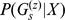 of genotype 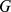 at site 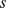 for individual 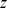 given the observed data 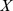 [11], [12]. The prior for obtaining these posteriors can be derived from an estimate of the allele frequency [11]. Similarly, empirical Bayes methods have been proposed to calculate the posterior probability 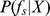 of the sample allele frequency 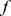 at site 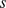 [11],[12]. 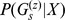 and 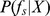 from simulated sequencing reads were computed using ANGSD software (http://popgen.dk/angsd).

Nucleotide diversity indices were calculated in a way that accounts for genotyping uncertainty, rather than strictly assigning individual genotypes. This probabilistic framework has been successfully adopted to estimate population genetics parameters from low sequencing depth data [11], [12], [14], [16], [17]. Throughout the study, the ancestral and derived allelic state were assumed to be known, and “allele frequency” refers to the frequency of the derived allele. All motivations are still valid under the folded site frequency spectrum (when ancestral and derived state are unknown).

Estimates of 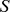 and 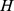 from sequencing data can be calculated as:
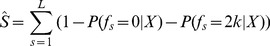
(3)where 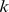 is the number of diploid individuals in the sample, and
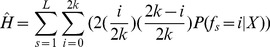
(4)where 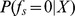, 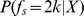, 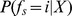 is the posterior probability of having 0, 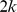, and 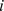 chromosomes with the derived allele at site 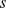, respectively [14].

Several experimental scenarios were explored by varying sequencing depth and sample size, while keeping their product (the total sequencing coverage) constant. 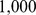, 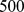, 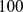, and 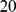 samples at 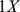, 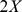, 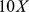, and 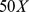, respectively were sub-sampled from the entire pool of 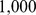 individuals. To assess the accuracy for estimating nucleotide variation under different experimental scenarios, the standardised bias between estimates obtained from known genotypes for all individuals and from unknown genotypes, for each window, was calculated as:
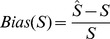
(5)and



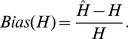
(6)Positive values of 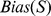 and 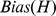 therefore indicate over-estimation of true values, while negative values indicate under-estimation. To directly quantify the effect of this bias on population genetics estimates, I identified windows showing extremely low or high values of 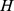 from the empirical distribution of all 100 windows for each experimental scenario. The number of correctly identified outliers using sequencing data, and the correlation between 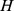 and 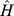 were used to measure estimation accuracy.

In the case of unknown genotypes, identifying variable sites in the sample can be achieved by detecting sites with a probability of being variable, calculated as 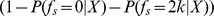 (see Equation 3), greater than a certain threshold. For each simulation, this threshold was dynamically chosen to minimise the difference between the number of true and estimated variable sites, in order to realise an optimal trade-off between SNP over-calling and SNP under-calling. Additional analyses were performed by setting the probability of being variable threshold to fixed values.

I evaluated the accuracy of SNP calling by computing False Positive (FP) and False Negative (FN) rates. Precision and Recall values were derived from these quantities. Precision is computed as the ratio of True Positive (TP) rates to (TP+FP), while Recall is the ratio of TP to (TP+FN). The average and standard deviation for Precision and Recall, and FP and FN rates, were calculated across all 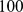 windows to inspect their distribution.

### Predicting Population Structure from Sequencing Data

I assessed the prediction accuracy of population structure under different experimental scenarios. Specifically, I compared the predicted population structure in the case of known genotypes from all individuals to the structure determined from the sequencing data for the entire pool of individuals, or a subset of it, at a fixed total sequencing depth. A total of 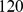 individuals with known genotypes were sampled from 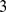 different sub-populations. Sample sizes of 40, 20, 4, and 2 individuals from each of the 3 populations, at 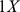, 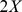, 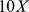, and 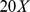 sequencing depth, respectively, were examined.

Principal Component Analyses (PCA) was used to inspect population genetics structure. The PCA is ultimately based on a covariance matrix of individual genotypes [40]. In the original latter approach, the denominator normalises the allele frequency variance. However, this normalisation over-weights low frequency variants and is therefore not suitable for NGS data, for which estimates of rare variants are usually less confident. Thus, the normalisation was not applied, without loss of generalisation throughout all analyses.

In cases where the genotypic covariance matrix had to be inferred directly from the sequencing data, previously proposed methods [17] were followed. Briefly, the posterior probability for the covariance matrix is approximated from the genotype posterior probabilities at each site for each individual. The covariance matrix is finally weighted by the probability of each site of being variable. This approach has been shown to perform well in cases of low sequencing depth and converges to standard genotype calling methods in cases of high sequencing depth [17]. Eigenvector decomposition of the covariance matrix is then performed to obtain the first 2 Principal Components (PCs). Given the simulation scheme used, these PCs contain the full information on population structure, while other PCs are likely to represent only stochastic noise. Procrustes Analysis techniques [41] were used to compare PCs obtained from the case of known genotypes and the case of unknown genotypes. Specifically, the PCs coordinates derived from unknown genotypes were rotated and scaled to minimise the distance to the corresponding coordinates of PCs computed from known genotypes.

A Support Vector Machine (SVM) algorithm was adopted to model and predict population structure over a 2-dimensional grid. SVM receives a training set of features and categories, and trains a machine to model the relationship between them. PCs coordinates were set as uncorrelated features and the population labelling at each set of coordinates as categories, and a model, for both the case of known genotypes and unknown genotypes, was estimated.

From these models, I predicted the population structure over a grid of 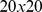 cells, as well as 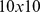 cells, from the model estimated from known and unknown genotypes separately. In other words, for each cell of the grid I predicted which population is located at that particular set of coordinates. I used the same grid, obtained by equally partitioning the PCs plane from known genotypes, for both models. Finally, the proportion of mislabelled populations between the model from known genotypes and from unknown genotypes over the entire grid was used as a measure of population structure prediction accuracy.

Programs to simulate sequencing data and to perform all described analyses are available at https://github.com/mfumagalli/ngsTools. All statistical analyses were performed in the R environment (www.r-project.org).

## Supporting Information

Figure S1
**Nucleotide diversity estimation with lower level of polymorphisms.** Bias in the estimate of the number of segregating sites (left panel) and the expected heterozygosity (right panel) under different experimental scenarios. Simulation were performed as described in Figure 1. I simulated 100 segments of 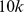 independent sites with the probability of each site being variable in the population equal to 0.01.(TIF)

Figure S2 **Nucleotide diversity estimation with lower sequencing error rate.** Bias in the estimate of the number of segregating sites (left panel) and the expected heterozygosity (right panel) under different experimental scenarios. Simulations were performed as described in Figure 1. The sequencing error rate was set to 0.005.
(TIF)

Figure S3 **Nucleotide diversity estimation under population size expansion.** Bias in the estimate of the number of segregating sites (left panel) and the expected heterozygosity (right panel) under different experimental scenarios. Simulations were performed as described in Figure 1. Populations were simulated under a size expansion model.
(TIF)

Figure S4 **Nucleotide diversity estimation with inbreeding.** Bias in the estimate of the number of segregating sites (left panel) and the expected heterozygosity (right panel) under different experimental scenarios. Simulations were performed as described in Figure 1. Genotypes were simulated assuming an individual inbreeding coefficient of 0.3.
(TIF)

Figure S5 **SNP calling accuracy with inbreeding.** False Positive and False negative rates for the identification of polymorphic sites under different experimental scenarios. Simulations were performed as described in Figure 2. Genotypes were simulated assuming an individual inbreeding coefficient of 0.3.
(TIF)

Figure S6 **SNP calling accuracy under population size expansion.** False Positive and False negative rates for the identification of polymorphic sites under different experimental scenarios. Simulations were performed as described in Figure 2. Populations were simulated under a size expansion model.
(TIF)

Figure S7 **SNP calling accuracy using a fixed cut-off.** False Positive and False negative rates in the identification of polymorphic sites under different experimental scenarios. Simulations were performed as described in Figure 2. Sites were identified as polymorphic if their probability of being variable was above 0.95.
(TIF)

Figure S8 **SNP calling accuracy using a fixed cut-off under population size expansion.** False Positive and False negative rates for the identification of polymorphic sites under different experimental scenarios. Simulations were performed as described in Figure 2. Sites were identified as polymorphic if their probability of being variable was above 0.95. Populations were simulated under a size expansion model.
(TIF)

Figure S9 **SNP calling accuracy using a fixed cut-off with inbreeding.** False Positive and False negative rates for the identification of polymorphic sites under different experimental scenarios. Simulations were performed as described in Figure 2. Sites were identified as polymorphic if their probability of being variable was above 0.95. Genotypes were simulated assuming an individual inbreeding coefficient of 0.3.
(TIF)

Figure S10 **SNP calling accuracy using a less stringent fixed cut-off.** False Positive and False negative rates for the identification of polymorphic sites under different experimental scenarios. Simulations were performed as described in Figure 2. Sites were identified as polymorphic if their probability of being variable was above 0.90.
(TIF)

Figure S11 **SNP calling accuracy using a more stringent fixed cut-off.** False Positive and False negative rates for the identification of polymorphic sites under different experimental scenarios. Simulations were performed as described in Figure 2. Sites were identified as polymorphic if their probability of being variable was above 0.99.
(TIF)

Figure S12 **SNP calling accuracy for common variants using a fixed cut-off.** False negative rates for the identification of polymorphic sites under different experimental scenarios. Simulations were performed as described in Figure 2. Sites were identified as polymorphic if their probability of being variable was above 0.95. Only sites with a true sample allele frequency greater than 0.01 were retained. Outliers are plotted as circles.
(TIF)

Figure S13 **SNP calling accuracy for common variants using a fixed cut-off under population size expansion.** False negative rates in the identification of polymorphic sites under different experimental scenarios. Simulations were performed as described in Figure S12. Populations were simulated under an expansion size model.
(TIF)

Figure S14 **SNP calling accuracy for common variants using a fixed cut-off with inbreeding.** False negative rates for the identification of polymorphic sites under different experimental scenarios. Simulations were performed as described in Figure S12. Genotypes were simulated assuming an individual inbreeding coefficient of 0.3.
(TIF)

Figure S15
**Population structure inference accuracy over a less dense grid.** Accuracy of population structure inference, measured as the proportion of the cells over a 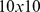 grid where sub-populations have been wrongly assigned compared to the case of known genotypes for all individuals (see Methods). Simulations were performed as described in Figure 3. Populations were simulated with high genetic subdivision (upper left panel, 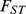 0.4 and 0.1), medium genetic subdivision (upper right panel, 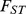 0.3 and 0.05), low genetic subdivision (lower left panel, 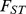 0.1 and 0.02). I also simulated 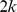 independent variable sites at medium genetic subdivision (lower right panel).(TIF)

Figure S16
**Population structure inference accuracy with all sites variable in the population.** Accuracy of population structure inference, measured as the proportion of the cells over a 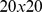 grid where sub-populations have been wrongly assigned compared to the case of known genotypes for all individuals (see Methods). Simulations were performed as described in Figure 3. I simulated 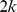 independent variable sites at medium genetic subdivision (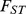 0.3 and 0.05).(TIF)

Table S1
**Power to detect outliers in the distribution of nucleotide diversity.** Accuracy of detecting outliers in the distribution of nucleotide diversity. Simulations were performed as described in Figure 1. The number of top and bottom (5 or 10 out of 100) windows from the distribution of 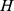 calculated from known genotypes that were correctly identified using sequencing data. Wilcoxon-test correlation between 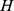 and 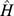 (see Methods) is also shown.(PDF)

Table S2
**SNP calling Precision and Recall with data filtering.** Precision and Recall values for detecting polymorphic sites at different scenarios of sequencing depth and sample size. Analyses were performed as described in Table 1. Sites with a total sequencing depth below the 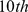 percentile were discarded.(PDF)

Table S3
**SNP calling Precision and Recall for the sample.** Precision and Recall values for detecting polymorphic sites at different scenarios of sequencing depth and sample size. Analyses were performed as described in Table 1. Accuracy was estimated by comparing true and estimated SNPs variable in the specific sample size, and not in the entire population of 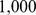 individuals.(PDF)
