# Supplementary material for: Assessing the Effect of Sequencing Depth and Sample Size in Population Genetics Inferences
Source: PLoS One. 2013 Nov 18;8(11):e79667. doi: 10.1371/journal.pone.0079667 (PMC3832539; doi:10.1371/journal.pone.0079667)
Supplement: Table S1 — Power to detect outliers in the distribution of nucleotide diversity. Accuracy of detecting outliers in the distribution of nucleotide diversity. Simulations were performed as described in Figure 1. The number of top and bottom (5 or 10 out of 100) windows from the distribution of calculated from known genotypes that were correctly identified using sequencing data. Wilcoxon-test correlation between and (see Methods) is also shown. (PDF) [file pone.0079667.s017.pdf]

Table S1 - Power to detect outliers in the distribution of nucleotide diversity

Accuracy of detecting outliers in the distribution of nucleotide diversity. Simulations were performed as described in Figure 1. The number of top and bottom (5 or 10 out of 100) windows from the distribution of H calculated from known genotypes that were correctly identified using sequencing data. Wilcoxon-test correlation between H and $\hat{H}$ (see Methods) is also shown.

Sequencing depth	Sample size	Bottom 5	Top 5	Bottom 10	Top 10	Correlation
1X	1,000	5	5	10	9	0.815
2X	500	5	5	10	8	0.958
10X	100	5	4	10	10	0.810
50X	20	3	5	7	9	0.277
