# Supplementary material for: Assessing the Effect of Sequencing Depth and Sample Size in Population Genetics Inferences
Source: PLoS One. 2013 Nov 18;8(11):e79667. doi: 10.1371/journal.pone.0079667 (PMC3832539; doi:10.1371/journal.pone.0079667)
Supplement: Table S2 — SNP calling Precision and Recall with data filtering. Precision and Recall values for detecting polymorphic sites at different scenarios of sequencing depth and sample size. Analyses were performed as described in Table 1. Sites with a total sequencing depth below the percentile were discarded. (PDF) [file pone.0079667.s018.pdf]

Table S2 - SNP calling Precision and Recall with data filtering

Precision and Recall values for detecting polymorphic sites at different scenarios of sequencing depth and sample size. Analyses were performed as described in Table 1. Sites with a total sequencing depth below the 10th percentile were discarded.

Sequencing depth	Sample size	Precision	Recall
1X	1,000	0.737(0.0466)	0.749 (0.0491)
2X	500	0.776 (0.0465)	0.773 (0.0473)
10X	100	0.776 (0.0482)	0.723 (0.0447)
50X	20	1 (0)	0.538 (0.0626)
