# Supplementary material for: Assessing the Effect of Sequencing Depth and Sample Size in Population Genetics Inferences
Source: PLoS One. 2013 Nov 18;8(11):e79667. doi: 10.1371/journal.pone.0079667 (PMC3832539; doi:10.1371/journal.pone.0079667)
Supplement: Table S3 — SNP calling Precision and Recall for the sample. Precision and Recall values for detecting polymorphic sites at different scenarios of sequencing depth and sample size. Analyses were performed as described in Table 1. Accuracy was estimated by comparing true and estimated SNPs variable in the specific sample size, and not in the entire population of individuals. (PDF) [file pone.0079667.s019.pdf]

Table S3 - SNP calling Precision and Recall for the sample

Precision and Recall values for detecting polymorphic sites at different scenarios of sequencing depth and sample size. Analyses were performed as described in Table 1. Accuracy was estimated by comparing true and estimated SNPs variable in the specific sample size, and not in the entire population of $1,000$ individuals.

Sequencing depth	Sample size	Precision	Recall
1X	1,000	0.737(0.0437)	0.749 (0.0472)
2X	500	0.832 (0.0428)	0.828 (0.0453)
10X	100	0.982 (0.0152)	0.981 (0.0186)
50X	20	1 (0)	1 (0)
